# Draft Genome Sequence of *Citrobacter freundii* Strain A47, Resistant to the Mycotoxin Deoxynivalenol

**DOI:** 10.1128/genomeA.00019-17

**Published:** 2017-03-16

**Authors:** Rafiq Ahad, Ting Zhou, Dion Lepp, K. P. Pauls

**Affiliations:** a Guelph Food Research Center, Agriculture Agri-Food Canada, Guelph, Ontario, Canada; b Department of Plant Agriculture, University of Guelph, Guelph, Ontario, Canada

## Abstract

Here, we present the draft genome sequence of *Citrobacter freundii* strain A47 with a length of 4,878,242 bp, which contains 4,357 putative protein coding genes, including 270 unique genes. This work is expected to assist in obtaining novel gene(s) that code for deoxynivalenol (DON) de-epoxidation enzyme(s).

## GENOME ANNOUNCEMENT

Agrifood commodities are often contaminated with hazardous mycotoxins such as deoxynivalenol (DON), produced by toxigenic *Fusarium* species (1–3). A potential environmentally friendly and effective method for addressing the problem may be the removal of the mycotoxin via enzymatic de-epoxidation (4, 5). In previous work toward this goal (6), a Gram-negative bacterial strain, *Citrobacter freundii* A47, was isolated from an enriched microbial culture that showed de-epoxidation activity at high concentrations of DON (500 µg/mL). The high level of DON de-epoxidation activity observed for *C. freundii* A47 led us to sequence its genome as it may contain DON de-epoxidation genes and enzymes.

The genome of *C. freundii* A47 was sequenced using an Illumina HiSeq 2000 platform at the Beijing Genomics Institute (BGI), Hong Kong. A 500 bp library was produced and sequenced with 90-bp paired-end reads. The raw data was filtered by removing >10% reads that include *N*’s or low complexity reads, low quality (≤Q20) bases, and adapter contamination, generating 126,619,724 filtered reads. Quality assurance was done by analysis of G+C content, depth correlation, and K-mer values. The cleaned short reads (180 bp) were assembled using SOAPdenovo v2. The assembly generated 439 contigs (*N*_50_ value: 29,222 bp) and 79 scaffolds (*N*_50_ value: 325,726 bp). The assembly represents a 107× coverage of total scaffolds with a total sequence length of 4,878,242 bp. The genome has an overall G+C content of 52%. The genome consists of an 88% coding sequence that represents 4,357 protein coding genes.

Since the DON de-epoxidation reaction is a reduction, the genome of *C. freundii* A47 was searched for reductase genes that are not present in closely related *Citrobacter* species that do not show de-epoxidation activity (7, 8). The analysis was done by all-versus-all reciprocal BLAST comparisons of coding sequences of strain A47 and genomes of five *Citrobacter* species in NCBI GenBank that are not expected to have DON de-epoxidation genes. The analysis was based on at least 30% amino acid identity and >70% coverage of the total length of the gene, using the OrthoMCL program (9). The comparative genome analysis resulted in the identification of 270 unique protein coding or hypothetical genes, including eight reductases as potential DON de-epoxidation genes in *C. freundii* A47. The accession numbers of the protein coding genes for reductases in the NCBI database are OCF82123.1, OCF82124.1, OCF80241.1, OCF83243.1, OCF83247.1, OCF83248.1, OCF81253.1, and OCF82809.1. Functional analyses of the proteins encoded by these unique reductase genes may lead to the identification of genes encoding DON de-epoxidizing enzymes. Subsequent biotechnological utilization of these genes could contribute to a safer supply of foods and feeds that are devoid of DON.

### Accession number(s).

This whole-genome shotgun project has been deposited in NCBI GenBank under the accession no. LNFS00000000. The version described in this paper is the first version, LNFS00000000.1.
